# Sex as a Moderator Between Parent Ratings of Executive Dysfunction and Social Difficulties in Children and Adolescents with Autism Spectrum Disorder

**DOI:** 10.1007/s10803-022-05629-5

**Published:** 2022-07-15

**Authors:** Tonje Torske, Terje Nærland, Daniel S. Quintana, Ruth Elizabeth Hypher, Anett Kaale, Anne Lise Høyland, Sigrun Hope, Jarle Johannessen, Merete G. Øie, Ole A. Andreassen

**Affiliations:** 1 Division of Mental Health and Addiction, Vestre Viken Hospital Trust, Postboks 800, 3004 Drammen, Norway; 2 Department of Psychology, University of Oslo, Oslo, Norway; 3 NevSom Department of Rare Disorders and Disabilities, Oslo University Hospital, Oslo, Norway; 4 K.G. Jebsen Center for Neurodevelopmental Disorders, University of Oslo, Oslo, Norway; 5 NORMENT, University of Oslo and Oslo University Hospital, Oslo, Norway; 6 Division of Mental Health and Addiction, Oslo University Hospital, Oslo, Norway; 7 Department of Special Needs Education, University of Oslo, Oslo, Norway; 8 Faculty of Medicine and Health Sciences, Department of Mental Health, Regional Centre for Child and Youth Mental Health and Child Welfare, Norwegian University of Science and Technology, Trondheim, Norway; 9 Department of Pediatrics, St. Olav Hospital, Trondheim University Hospital, Trondheim, Norway; 10 Department of Neurohabilitation, Oslo University Hospital, Oslo, Norway; 11 Department of Medicine, University of Oslo, Oslo, Norway; 12 Research Department, Innlandet Hospital Trust, Lillehammer, Norway; 13 Division of Pediatric and Adolescent Medicine, Department of Clinical Neurosciences for Children, Oslo University Hospital, Oslo, Norway

**Keywords:** Autism spectrum disorder, Executive function, Sex differences, Behavior rating inventory of executive function (BRIEF), Autism diagnostic interview revised (ADI-R)

## Abstract

Girls and boys might differ in autistic symptoms and associated cognitive difficulties such as executive function (EF). We investigated sex differences in the relationship between parent rated EF and autistic symptoms in 116 children and adolescents (25 girls) aged 5–19 years with an intelligence quotient above 70 and an autism spectrum disorder (ASD) diagnosis. They were rated with the behavior rating inventory of executive function (BRIEF) and the autism diagnostic interview revised (ADI-R). We found a positive association between EF and the ADI-R domains of reciprocal social interaction (*p* < 0.001) and communication (*p* = 0.001) in girls, while these relationships were small and non-significant in boys. Our results provide a greater understanding of the sex-specific characteristics of children and adolescents with ASD.

**Supplementary Information:**

The online version contains supplementary material available at 10.1007/s10803-022-05629-5.

Autism spectrum disorder (ASD) is overrepresented in males compared to females. Therefore, research on females with ASD has been limited, and most of the literature on ASD is based on boys and young men (Lai et al., 2012). However, there is a growing interest and need for a better understanding of sex differences in ASD, which is reflected by an increased research focus on females with ASD (Halladay et al., 2015; Lai et al., 2015; Werling & Geschwind, 2013). Autistic symptoms seem to be less apparent in girls than boys. This phenomenon might be due to girls learning compensatory behaviors and skills to mask their social challenges (Dean et al., 2017; Lai et al., 2017b) and that parents, teachers, and clinicians are less able to recognize autistic symptoms in girls (Ratto et al., 2018). Girls within populations-based samples with high levels of autistic-like traits tend to have better social skills and less behavioral problems than boys with comparably high levels of ASD traits, which might make it harder to recognize their autistic characteristics (Dworzynski et al., 2012). Females with ASD also tend to have less restricted and repetitive behavior and interests (RRB) compared to males with ASD (Lai et al., 2015; Van Wijngaarden-Cremers et al., 2014). Further, females who reach the diagnostic threshold of ASD often have lower intelligence scores and more cognitive and behavioral problems than boys with ASD (Dworzynski et al., 2012). An important factor in the skewed prevalence ratio in ASD seems to be related to cognitive level as lower intelligence quotient (IQ) is associated with a lower male-to-female ratio (Lehnhardt et al., 2016; Loomes et al., 2017; Werling & Geschwind, 2013).

## Executive Dysfunction in Individuals with ASD

Together with the theory of mind hypothesis and the weak central coherence theory, the executive dysfunction hypothesis is one of the central cognitive theories that seeks to explain the core difficulties in ASD (Pellicano, 2011). Traditionally, executive function (EF) deficits, and particularly inflexibility, have been associated with restricted and repetitive behavior (RRB) in ASD (Hill, 2004; Lopez et al., 2005). Others have also related EF deficits to difficulties with social function and communication (Chouinard et al., 2019; Kenworthy et al., 2009). Studies investigating the relationship between EF and autistic symptoms have focused on specific subdomains of EF examined mainly by neuropsychological tests (Bolte et al., 2011; Lehnhardt et al., 2016; Lemon et al., 2011; Nyden et al., 2000). Recent meta-analyses confirm that both girls and boys with ASD perform worse on EF tasks than typically developing (TD) controls, on average (Demetriou et al., 2017; Lai et al., 2017a). EF comprises several components including inhibition, working memory, flexibility, emotional control, initiation, planning, organization, monitoring, and self-control (Hill, 2004; Miyake et al., 2000). These components enable the individual to disengage from the present context to work towards future goals. EF impairments have been associated with many psychiatric disorders including attention deficit/hyperactivity disorder (ADHD) and ASD (Dajani et al., 2016). Demetriou et al. (2017) found consistent evidence of an overall moderate effect size (Hedges’ *g* = 0.48) of executive dysfunction in ASD, and that these deficits are relatively stable across development, with few differences across subdomains (Demetriou et al., 2017). In a meta-analysis that also included children and adolescents with ASD and comorbid ADHD, Lai et al. (2017a) confirmed that children with ASD tend to have executive dysfunction with small-to-moderate effect sizes (Hedges’ *g* = 0.41–0.67), and that this was not solely accounted for by the effect of comorbid ADHD or general cognitive abilities (Lai et al., 2017a).

Since some EF difficulties may not be observable in a laboratory setting, informant based measures and questionnaires like the behavior rating inventory of executive function (BRIEF) can add valuable information about the relationship (Kenworthy et al., 2008). The BRIEF was found to be a better clinical marker of EF difficulties than performance based tests in individuals with ASD (Demetriou et al., 2017). This is probably because it can be difficult to generalize from EF assessed in highly structured laboratory settings, and that questionnaires regarding everyday functioning have a higher ecological validity and thus also a better clinical utility than neuropsychological tests (Demetriou et al., 2017; Kenworthy et al., 2008). In addition, intelligence and age are factors that might influence EF in children with ASD, and therefore important to consider when investigating the relationship between EF and autistic symptoms (Van Eylen et al., 2015).

## Sex Differences in EF in Individuals with ASD

Some researchers have found sex differences in EF in TD individuals (Kiep & Spek, 2017). However, a recent review of sex differences in EF based on both human studies and animal research concluded that there is limited support for substantial sex differences (Grissom & Reyes, 2019). Still, some studies have indicated that females with ASD have more impairment in EF compared to males (Lemon et al., 2011). In a relatively small group of participants, Lemon et al. (2011) found that girls with ASD showed poorer response inhibition than TD girls and boys with ASD. Others have reported that females with ASD outperform males on executive tasks related to processing speed and verbal fluency (Bolte et al., 2011; Lehnhardt et al., 2016). Kiep & Spek (2017) compared adults with ASD and TD on a variety of neuropsychological tasks assessing EF. They found sex differences in both TD and ASD on several EF tasks. However, they highlighted that IQ level and the type of EF assessment influenced the results, and they could not pinpoint any sex specific cognitive profiles. Furthermore, the variation within is greater than the variation between the sexes in EF, but males and females might have different strategies and developmental trajectories which influence their EF performance (Grissom & Reyes, 2019). White and colleagues (White et al., 2017) reported a correlation between EF difficulties in everyday life and decreased adaptive ability in both boys and girls with ASD. However, girls had more EF difficulties on the BRIEF and more difficulties on the Daily Living Skills domain on the Vineland Adaptive Behavior Scales than boys.

## Sex as a Moderator Between EF Deficits and ASD Symptoms

There is a growing interest for investigating possible sex differences in autism when it comes to topics like mechanisms, prevalence, clinical characteristics, the validity of diagnostic instruments for both sexes and treatments effects*.* Although studies have shown a relationship between key ASD symptomatology and EF, there are few studies focusing on if *sex* moderates the relationship, and the findings have been inconsistent. In an earlier study we found some preliminary evidence for a stronger positive association between EF deficits and social problems in girls than in boys with ASD (Torske et al., 2017). Therefore, we wanted to investigate this in more depth in a larger sample by examining how different aspects of autistic symptomatology are related to EF using a summary score from parent rated EF, namely the global executive composite (GEC) from the BRIEF. Earlier, we also investigated the relationship between polygenic risks scores and EF in children and adolescents with ASD (Torske et al., 2019). However, we did not find any sex differences in the latter study.

The main aim of the current study was to investigate if sex moderates the relationship between parent-rated EF in everyday life and autistic symptomology measured by parent interviews. We also explored the influence of age, IQ, and ADHD diagnosis on these relations. In line with our previous findings, we anticipated that there might be a closer association between EF and autism symptomatology in girls than in boys. Furthermore, we expected EF to be related to all aspects of ASD symptomatology (social interaction, communication and RRB). Improving knowledge about potential sex differences in the relationship between EF and autistic symptoms is important for understanding how sex impacts clinical manifestations and diagnosis, and might have implications for the development of differentiated interventions for girls and boys with ASD.

## Methods

### Participants

Participants were recruited from Norwegian health services specializing in the assessment of ASD and other neurodevelopmental disorders. The study was part of the national BUPGEN network (Grove et al., 2019) and the methods are described previously in Torske et al. (2017). The current sample consisted of 25 girls and 91 boys with ASD who were recruited between 2013 and May 2018 and assessed at age 5–19 years. Fifteen of the children (2 girls, 13 boys) were diagnosed with childhood autism, 9 (2 girls, 7 boys) with atypical autism, 57 (14 girls, 43 boys) with Asperger syndrome and 35 (7 girls, 28 boys) with unspecified pervasive developmental disorder (PDD-NOS). Because ASD and ADHD often co-occur (Lord et al., 2018), the current study also included children with ASD and comorbid ADHD.

The male:female ratio was 3.6:1. In total, 40 children (34.5%) had a comorbid disorder of ADHD, and the male:female ratio was 7:1 in the ADHD group. All participants had a full-scale intelligence quotient (IQ) ≥ 70 based on a standardized Wechsler’s test (Full-scale IQ). We did not use any standardized assessment to determine if the participants spoke Norwegian fluently. However, the psychologist who administered the intelligence test carefully evaluated the language fluency of the children in relation to the test session and made sure that they understood the instructions for the test.

### Clinical Assessment

Participants were assessed by a team of experienced clinicians (clinical psychologists and/or child psychiatrists and educational therapists). Diagnostic conclusions were best-estimate clinical diagnoses derived from tests, interview results, and observations. All diagnoses were based on the International statistical classification of diseases and related health problems 10th revision (ICD-10) (World Health Organization, 1992) criteria, and the autistic symptoms were evaluated using the autism diagnostic observation schedule (ADOS-G and ADOS-2) modules 2, 3 or 4 (Lord et al., 2000, 2012) and/or autism diagnostic interview-revised (ADI-R) (Rutter et al., 2003). In addition, the assessment included a full medical and developmental history, physical examination, IQ assessment and parent ratings of EF. The ADHD diagnosis were based on a thorough diagnostic process including detailed clinical examination, cognitive tests, and questionnaires. However, unfortunately, we did not have access to severity score of ADHD symptomatology for this sample.

### Measures

#### Autistic Symptoms

Autism diagnostic interview-revised (ADI-R) diagnostic algorithm was used to evaluate autistic symptoms. The ADI-R is a clinical diagnostic tool based on a comprehensive interview with parents or primary caregivers of the child/ adolescent (Lord et al., 1994). The interview consists of 93 questions, and a predetermined number of these scores are used in a diagnostic algorithm. The interview and scoring follow standardized procedures, and the interviewer records and codes the informant’s responses. The algorithm is divided into three functional domains based on the diagnostic criteria: A = Reciprocal Social Interaction, B = Communication, C = Restricted, Repetitive, and Stereotyped Behavior. Higher scores indicate that an individual has a greater number of items representing core ASD deficits and/or more severe symptoms (Gotham et al., 2009). All participants had sufficient verbal skills to be considered as “verbal” according to the ADI-R manual, and therefore the algorithm for verbal children was used. We used the Norwegian translation of the ADI-R (Rutter et al., 2016).

#### Executive Function (EF)

In order to report EF difficulties, parents completed the parent version of the BRIEF for children and adolescents aged 5–18 years (Gioia et al., 2000). The questionnaire includes 86-items where parents report the child’s everyday EF in the home and school environments (Gioia et al., 2000). The BRIEF contains eight scales that are grouped in a behavioral regulation index (BRI): Inhibit, Shift and Emotional Control, and a metacognition index (MI): Initiate, Working Memory, Plan/Organize, Organization of Materials and Monitor. *T-scores* of ≥ 65 are considered to represent a clinically significant score. The global executive composite (GEC), which is used in the present study, is a summary score that incorporates all eight clinical scales. The GEC has high reliability in both standardized and clinical samples (Cronbach´s alpha = 0.80–0.98). The current study used the Norwegian version of the parent rating form, which has been reported to have high internal consistency (Cronbach´s alpha = 0.76–0.92) (Fallmyr & Egeland, 2011). Similar levels are described for the English version (Cronbach´s alpha = 0.80–0.98) (Gioia et al., 2000)*.*

#### Intelligence Quotient (IQ)

IQ was assessed using age-appropriate full-scale Wechsler tests of intelligence (Wechsler, 2002, 2003, 2008)*.* Most of the participants were assessed with Wechsler Intelligence Scale for Children-Forth Edition, and some were assessed with Weschler Preschool and Primary Scale of Intelligence-Third Edition or Wechsler Adult Intelligence Scale-Forth Edition. We used the Norwegian versions of the Wechsler tests, which have Norwegian and/or Scandinavian norms (Weschler, 2008, 2009, 2011).

### Statistical Analyses

Analyses were conducted using the R statistical environment (version 4.2.0) using the “jmv” (Version 2.3.4; (Selker et al., 2017)). The FUZZY extension command in SPSS was used to generate a closely matched sample of boys and girls on age and IQ. Checks for multicollinearity, homoscedasticity, influential outliers, homogeneity of variance, and normality of residuals suggested that assumptions for analyses were met. Statistical significance was set at *p* < 0.05 and adjusted according to number of comparisons. We provide justifications below for how we adjusted tests for multiple comparisons to control the Type-I error rate. Conventional values were used for interpreting effect sizes (Effect size values of 0.2, 0.5, and 0.8, were considered small, medium, and large effects, respectively (Cohen, 1988)).

We have used T-scores from the BRIEF in all the analyses. T-scores can provide clinical meaning to scores, since age and sex can affect what is considered “typical” behavior. Furthermore, we find it important to use t-scores because deviations from TD is important, particularly as girls and boys develop in different ways and parents might have different expectations between girls and boys. In general, age influences t-scores more than sex differences. For example, a t-score of 69 on GEC corresponds to a raw score of 163–164 for girls aged 8–10 years and a raw score of 165–166 for boys aged 8–10 years.

Welch’s t-tests were conducted to investigate sex differences in ADI-R and BRIEF scores. We used the Welch’s t-test because it is recommended as a default instead of the Student´s t-test, even if variances are equal (Delacre et al., 2017). As we were examining a series of tests and hypothesizing that these groups were not significantly different, we adjusted for 6 tests, ADI-R A, ADI-R B, ADI-R C, BRIEF GEC, MI and BRI (critical *p*-value = 0.008); (Armstrong, 2014; Perneger, 1998), with values less than 0.05 considered on the border of statistical significance (i.e., Bonferroni correction). A chi-squared statistic was calculated to assess the frequency distribution of comorbid ADHD between sexes. For the t-tests, Glass’ delta—which is unaffected by unequal variances—was used as a measures of effect size. Due to the relatively large age range, we also present sex differences in ADI-R and BRIEF scores separately for girls and boys pre- and post-puberty (11 years and younger and 12 years and older) (See Supplementary Tables S8 and S9).

To investigate the association between ADI-R sub-scores (i.e., reciprocal social interaction, communication, and RRB) and EF (BRIEF GEC), we first calculated a Pearson correlation coefficient. To investigate the impact of covariates (i.e., sex, IQ, age, ADHD, and a sex * EF interaction) on the association between ADI-R sub-scores and BRIEF GEC, we fitted a series of nested multiple regression models and then compared the fit of these models by calculating Akaike information criterion (AIC) values and *F*-ratios for model change. For sex, “male” was the reference category, and for the ADHD variable, “no ADHD diagnosis” was the reference category. Lower AIC values are indicative of better model fit. As we were interested in three sub-scores from the ADI-R for these multiple regression models, we adjusted the critical value for 3 tests (ADI-R A, ADI-R B, ADI-R C) (critical *p*-value = 0.017), with values less than 0.05 considered on the border of statistical significance for the purposes of these analyses (i.e., Bonferroni correction). Although this is an arbitrary cutoff for values considered to be on the border of statistical significance, we chose 0.05 as this is the value traditionally used when analyses are not corrected for multiple comparisons. To generalise the regression results beyond the given samples, robust regression was performed in the event of non-normally distributed standardized residuals via bootstrapping with 2000 samples. We obtained bootstrapped 95% confidence intervals for the model intercept and slopes and compared these with the confidence intervals from the original model when the standardized residuals from models were not normally distributed. Confidence intervals for the intercept and slopes of these models were similar to a bootstrapped model, indicating that there were no considerable problems with non-normal distribution of residuals in the model (See Supplementary Tables S5–S7 for details). To examine the impact of more closely matched boys and girls on age and IQ, the same model fit and comparison procedure was performed on a subset of the sample, which was generated using the FUZZY extension command in SPSS. These analyses can be found in the supplement section. We allowed cases to be matched on age within 2 years and total IQ within 10 points. Three girls had missing full-scale IQ data, so the 22 girls with no missing values were matched to 44 boys (Supplementary Table S1). Analyses of the BRIEF subdomains behavioral regulation index (BRI) and metacognition index (MI) for the total sample are shown in Supplementary Tables S10–S15.

## Results

### Sex Differences in Age, IQ, ADI-R Scores, and BRIEF Scores

There were no statistically significant differences between sexes (critical alpha adjusted to *p* = 0.008) in any of the ADI-R domains, BRIEF GEC, full-scale IQ, or age (Table 1). However, there were tendencies for girls to be slightly older (*p* = 0.029), have some more difficulties on the BRIEF index MI (*p* = 0.045) and to have less difficulties with the ADI-R C domain restricted and repetitive behavior (*p* = 0.038) than the boys, but these sex differences did not reach the adjusted significance level. We also found no significant differences between girls and boys in any of the ADI-R domains, BRIEF index scores, full-scale IQ, or age when we split the sample into pre-puberty and post-puberty groups (Supplementary Tables S8 and S9).

**Table 1:** Age, IQ, BRIEF and ADI-R scores for girls and boys with ASD (N = 116)

Scale	Girls		Boys		df	*p-value*	Glass’ delta
	Mean (SD)	n	Mean (SD)	n			
Age	12.0 (3.1)	25	10.4 (3.2)	91	39.0	0.029	− 0.50
Full-scale IQ	93.5 (9.3)	22	95.6 (13.1)	80	46.5	0.386	0.16
BRIEF Global Executive Composite (GEC)	69.4 (10.1)	25	67.2 (10.8)	91	40.3	0.349	− 0.20
BRIEF Behavioral Regulation Index (BRI)	67.6 (14.6)	25	68.0 (11.8)	86	33.7	0.917	0.03
BRIEF Metacognition Index (MI)	68.6 (8.3)	25	64.5 (11.0)	91	49.9	0.045	− 0.37
ADI-R (A) Reciprocal Social Interaction domain	11.8 (6.1)	25	11.7 (5.1)	91	33.5	0.945	− 0.02
ADI-R (B) Communication domain	8.8 (5.2)	24	9.2 (4.3)	87	32.4	0.715	0.10
ADI-R (C) Restricted, repetitive and stereotyped behavior domain	2.4 (2.1)	24	3.4 (2.2)	88	38.4	0.038	0.47

Welch’s t-tests were conducted for age, IQ, BRIEF and ADI-R comparisons between sexes BRIEF scores are reported as T scores (M = 50, SD = 10) and ADI-R scores are reported as domain scores from the diagnostic algorithm

*IQ* intelligence quotient, *BRIEF* behavior rating inventory of executive functions, *ADI-R* autism diagnostic interview-revised

^*^p = 0.008

There was no significant diffference in the proportion of boys and girls with comorbid ADHD (χ^2^ = 2.96, *p* = 0.09).

### The Association Between Reciprocal Social Interaction and EF

There was a statistically significant correlation (adjusted critical alpha = 0.017) between reciprocal social interaction and EF (r = 0.31, *p* < 0.001), as indexed by scores on the ADI-R-A and BRIEF GEC, respectively. We fitted three nested linear regression models to assess the role of covariates (i.e., sex, IQ, age, and ADHD diagnosis) and the interaction of sex and EF on the relationship between reciprocal social interaction and BRIEF GEC (Table 2). The first model, which included sex, IQ, age, and ADHD diagnosis, was not statistically significant (*p* = 0.49). The second nested model, which added BRIEF GEC, was on the border of our adjusted statistical significance threshold (*p* = 0.04). The second model (AIC = 630.9) was a significantly better fit of the data than the first model (AIC = 637.4; F(1, 96 = 8.38, *p* = 0.005), indicating that EF is related to reciprocal social interaction, over and above the main effects of sex, IQ, age, and ADHD diagnosis. The third nested model, which added the interaction of BRIEF GEC and sex, significantly predicted social interaction (*p* = 0.001). In this model, BRIEF GEC, sex, and their interaction provided a statistically significant contribution (Table 2). The third model (AIC = 619.7), which included a sex * BRIEF GEC interaction term, was a significantly better model for the data than the second model, which only included main effects (AIC = 630.9; F(1, 95) = 13.15, *p* < 0.001). Simple slopes analysis revealed a statistically significant slope for females (0.47, SE = 0.1, t = 4.63, *p* < 0.0001) but not for males (0.05, SE = 0.05, t = 0.95, *p* = 0.35). In other words, a one-point increase in ADI-R-A score is associated with a 0.47 BRIEF GEC score increase in females, but only a 0.05 BRIEF GEC score increase in males.

**Table 2:** Nested hierarchical model summary: reciprocal social interaction domain

ADI-R A	R^2^	B	SE B	95% CI	*p*
*Model 1*	0.03				0.486
Constant		19.98	5.20	[9.65, 30.30]	< .001*
Sex		− 0.13	1.32	[− 2.75, 2.49]	0.923
IQ		− 0.07	0.04	[− 0.16, 0.02]	0.104
ADHD diagnosis		− 1.26	1.16	[− 3.56, 1.04]	0.279
Age		− 0.08	0.17	[− 0.42, 0.25]	0.618
*Model 2*	0.11				0.041
Constant		10.68	5.95	[− 1.14, 22.49]	0.076
Sex		− 0.55	1.28	[− 3.09, 1.99]	0.667
IQ		− 0.07	0.04	[− 0.15, 0.01]	0.106
ADHD diagnosis		− 1.88	1.14	[− 4.13, 0.38]	0.102
Age		− 0.07	0.16	[− 0.40, 0.25]	0.650
BRIEF GEC		0.14	0.05	[0.04, 0.24]	0.005*
*Model 3*	0.22				< .001*
Constant		44.69	10.93	[22.99, 66.39]	< .001*
Sex		− 29.20	7.99	[− 45.07, − 13.34]	< .001*
IQ		− 0.07	0.04	[− 0.15, 0.01]	0.100
ADHD diagnosis		− 1.04	1.10	[− 3.21, 1.14]	0.345
Age		− 0.05	0.15	[-0.36, 0.25]	0.743
BRIEF GEC		− 0.37	0.15	[− 0.66, − 0.07]	0.015*
BRIEF GEC * Sex		0.42	0.11	[0.19, 0.65]	< .001*

*ADI-R* autism diagnostic interview- revised, diagnostic algorithm, *A* reciprocal social interaction domain, *B* communication domain, *C* restricted, repetitive and stereotyped behavior domain, *ADHD* attention deficit/hyperactivity disorder, *IQ* intelligence quotient, *BRIEF_GEC* behavior rating inventory of executive function, global executive composite, *B* unstandardized regression coefficients, *CI* confidence interval

^*^p = 0.017

**Fig. 1:**
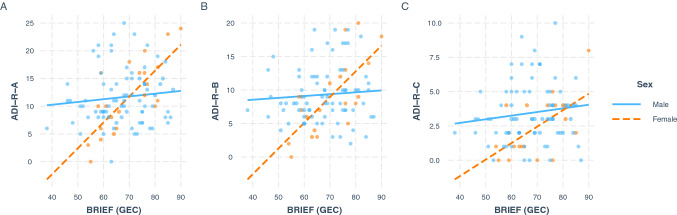
Simple slopes plots of the interaction between sex and executive function for the prediction of ADI-R subscores. ADI-R autism diagnostic interview- revised, diagnostic algorithm.** A** reciprocal social interaction domain,** B** communication domain,** C** restricted, repetitive and stereotyped behavior domain. BRIEF_GEC behavior rating inventory of executive function, global executive composite. BRIEF scores are reported as T scores (M = 50, SD = 10) and ADI-R scores are reported as domain scores from the diagnostic algorithm

The same model fit and comparison procedure on subset of participants more closely matched on age and IQ revealed similar results (Supplementary Table S2) (Fig. 1).

### The Association Between Communication and EF

There was a statistically significant correlation (adjusted critical alpha = 0.017) between communication and EF (r = 0.33, *p* < 0.001), as indexed by scores on the ADI-R-B and BRIEF GEC, respectively. We fitted three nested linear regression models to assess the role of covariates and the interaction of sex and EF on the relationship between ADI-R B and BRIEF GEC (Table 3). The first model, which including sex, IQ, age, and ADHD diagnosis, was not statistically significant (*p* = 0.84). Although the second nested model was also not statistically significant (*p* = 0.20), BRIEF GEC provided a contribution that was on the border of statistical significance (*p* = 0.02). This second model (AIC = 577.3) was a better fit of the data than the first model (AIC = 581.5; F(1, 92) = 5.98, *p* = 0.02), indicating that EF is related to communication, over and above the main effects of sex, IQ, and ADHD diagnosis. However, this effect was on the border of statistical significance (*p* = 0.02) and needs to be validated in future studies. The third nested model, which added the interaction of BRIEF GEC and sex, significantly predicted communication (*p* = 0.004). In this model, BRIEF GEC, sex, and their interaction provided a statistically significant contribution (Table 3). The third model (AIC = 566.9), which included a sex * BRIEF GEC interaction term, was a significantly better model for the data than the second model, which only included main effects (AIC = 577.3; F(1, 91) = 12.27, *p* = 0.001). Simple slopes analysis revealed a statistically significant slope for females (0.38, SE = 0.09, t = 4.29, *p* < 0.0001) but not for males (0.03, SE = 0.05, t = 0.61, *p* = 0.55). In other words, a one-point increase in ADI-R-B score is associated with a 0.38 BRIEF GEC score increase in females, but only a 0.03 BRIEF GEC score increase in males. The same model fit and comparison procedure on subset of participants more closely matched on age and IQ revealed similar results (Supplementary Table S3).

**Table 3:** Nested hierarchical model summary: Communication domain

ADI-R B	R^2^	B	SE B	95% CI	*p*
*Model 1*	0.01				0.843
Constant		12.86	4.47	[3.98, 21.74]	0 .005*
Sex		− 0.42	1.14	[− 2.68, 1.85]	0.717
IQ		− 0.04	0.04	[− 0.11, 0.04]	0.357
ADHD diagnosis		0.44	1.01	[− 1.57, 2.45]	0.664
Age		0.01	0.15	[− 0.28, 0.30]	0.963
*Model 2*	0.07				0.200
Constant		6.11	5.16	[− 4.14, 16.35]	0.239
Sex		− 0.76	1.12	[− 2.99, 1.47]	0.500
IQ		-0.03	0.04	[− 0.11, 0.04]	0.378
ADHD diagnosis		− 0.01	1.00	[− 2.01, 1.98]	0.990
Age		0.01	0.14	[− 0.27, 0.29]	0.949
BRIEF GEC		0.10	0.04	[0.02, 019]	0.016*
*Model 3*	0.18				0.004*
Constant		35.20	9.62	[16.08, 54.31]	< 0.001*
Sex		− 25.36	7.10	[− 39.47, − 11.26]	< .001*
IQ		− 0.03	0.03	[− 0.10, 0.04]	0.404
ADHD diagnosis		0.75	0.97	[− 1.18, 2.68]	0.439
Age		0.01	0.13	[− 0.25, 0.28]	0.916
BRIEF GEC		− 0.33	0.13	[− 0.59, − 0,07]	0.013*
BRIEF GEC* Sex		0.36	0.10	[0.15, 0.56]	< .001*

*ADI-R* autism diagnostic interview- revised, diagnostic algorithm, *A* reciprocal social interaction domain, *B* communication domain, *C* restricted, repetitive and stereotyped behavior domain, *ADHD* attention deficit/hyperactivity disorder, *IQ* intelligence quotient, *BRIEF_GEC* behavior rating inventory of executive function, global executive composite, *B* unstandardized regression coefficients, *CI* confidence interval

*p* = 0.017

### The Association Between Restricted, Repetitive and Stereotyped Behavior and EF

The correlation between restricted, repetitive and stereotyped behavior and EF, as indexed by scores on the ADI-R–C and BRIEF GEC respectively, was on the border of the adjusted critical alpha (r = 0.22, *p* = 0.019; adjusted critical alpha = 0.017). We fitted three nested linear regression models to assess the role of covariates and the interaction of sex and EF on the relationship between repetitive behavior and EF (Table 4). The first model, which including sex, IQ, age, and ADHD diagnosis, was not statistically significant (*p* = 0.43). Nor was the second nested model which added BRIEF GEC (*p* = 0.12). This second model (AIC = 439.9) was a better fit of the data than the first model (AIC = 443.1; F(1, 93) = 5.08, *p* = 0.03), but was on the border of statistical significance. The third nested model (adding the interaction of BRIEF GEC and sex) was not statistically significant (*p* = 0.06) (Table 4). The third model (AIC = 438.4) was a better fit of the data than the second model (AIC = 439.9), but this was not statistically significant (F(1, 92) = 3.3, *p* = 0.07). Simple slopes analysis revealed a statistically significant slope for females (0.12, SE = 0.05, t = 2.65, *p* = 0.01) but not for males (0.03, SE = 0.02, t = 1.15, *p* = 0.25). In other words, a one-point increase in ADI-R–C score is associated with a 0.12 BRIEF GEC score increase in females, but only a 0.03 BRIEF GEC score increase in males. The same model fit and comparison procedure on subset of participants more closely matched on age and IQ revealed similar results (Supplementary Table S4).

**Table 4:** Nested hierarchical model summary: Restricted, repetitive and stereotyped behavior domain

ADI-R C	R^2^	B	SE B	95% CI	*p*
*Model 1*	0.04				0.435
Constant		5.70	2.16	[1.41, 9.98]	0 .010*
Sex		− 1.00	0.55	[− 2.09, 0.10]	0.073
IQ		− 0.01	0.02	[− 0.05, 0.02]	0.478
ADHD diagnosis		− 0.39	0.48	[− 1.35, 0.57]	0.422
Age		0.01	0.07	[− 0.13, 0.15]	0.889
*Model 2*	0.09				0.118
Constant		2.69	2.50	[− 2.28, 7.65]	0.286
Sex		− 1.15	0.54	[− 2.23, − 0.07]	0.037
IQ		− 0.01	0.02	[− 0.05, 0.02]	0.506
ADHD diagnosis		− 0.58	0.48	[− 1.54, 0.37]	0.229
Age		0.01	0.07	[− 0.13, 0.15]	0.873
BRIEF GEC		0.05	0.02	[0.01, 0.09]	0.027
*Model 3*	0.12				0.061
Constant		10.33	4.88	[0.64, 20.01]	0.037
Sex		− 7.62	3.60	[− 14.77, -0.47]	0.037
IQ		− 0.01	0.02	[− 0.05, 0.02]	0.533
ADHD diagnosis		− 0.39	0.49	[− 1.35, 0.58]	0.428
Age		0.01	0.07	[− 1.12, 0.15]	0.857
BRIEF GEC		− 0.07	0.07	[− 0.20, 0.06]	0.309
BRIEF GEC * Sex		0.09	0.05	[− 0.01, 0.20]	0.072

*ADI-R* autism diagnostic interview- revised, diagnostic algorithm, *A* reciprocal social interaction domain, *B* communication domain, *C* restricted, repetitive and stereotyped behavior domain, *ADHD* attention deficit/hyperactivity disorder, *IQ* intelligence quotient, *BRIEF_GEC* behavior rating inventory of executive function, global executive composite, *B* unstandardized regression coefficients, *CI* confidence interval

^*^p = 0.017

## Discussion

The main aim of the current study was to investigate if sex moderates the relationship between parent-rated EF in everyday life and autistic symptomology measured by parent interviews. The main finding of the current study is that sex moderates the relationship between parent-reported EF in everyday life and social and communication difficulties in children and adolescents with ASD. We found a positive association between the BRIEF (GEC) scores and the ADI-R domains reciprocal social interaction and communication in girls, while these relationships were small and non-significant in boys. We did not find sex differences in the relationship between executive dysfunction and restricted and repetitive behaviors. Although the results were statistically significant, it is important to emphasize that the effect sizes were modest, and the confidence intervals were relatively wide. Together, these results may have implications for understanding the different clinical manifestations of ASD in girls and boys. Furthermore, it supports the notion that there may be different reasons for the behavioral problems related to ASD in girls and boys, with girls’ social and communicative challenges being more strongly related to EF deficits. There is some evidence that girls with ASD camouflage their ASD related difficulties to a larger extend than boys. Girls with executive dysfunction and ASD might experience a “double hit” which makes it harder for them to use compensatory techniques, and therefore will show more ASD related difficulties than girls with ASD and intact EF (Livingston & Happe, 2017). Others have also found the relationship between EF and social communication to be affected by sex both for typically developing children and for children with ASD (Chouinard et al., 2019; Dai et al., 2019). This could also help to develop sex-differentiated interventions.

Of note, we found evidence for a sex-specific relationship between parent rated EF deficits and difficulties in the domains social reciprocity and communication, but not for the relationship between EF deficits and RRB. This differs from previous studies, which found that EF difficulties were mainly related to RRB (Brunsdon & Happe, 2014; Lopez et al., 2005). However, these studies did not investigate the differences between girls and boys. On the other hand, Kenworthy and colleagues showed that EF deficits, measured with both performance tests and parental questionnaires, were related to all three components of the triad of impairment in ASD (Kenworthy et al., 2009). One possible explanation for the lack of a significant relationship between EF and RRB in our study might be the use of a general measure of EF, namely the Global Executive Composite from the BRIEF. In a recent paper by Faja & Darling (2019), they found the subscale Shift from the BRIEF to be a significant predictor of higher order RRB like circumscribed interests and ritualistic behavior (Faja & Nelson Darling, 2019). Therefore, it might be easier to find a relationship between RRB and EF when one has a narrower focus on both EF and RRB rather than investigating more general categories. This is also in line with Kiep and Spek’s results which, despite finding sex differences in specific tasks, could not identify sex specific cognitive profiles (Kiep & Spek, 2017).

We did not find any statistically significant sex differences in the total amount of difficulties with social reciprocity or communication (ADI-R A and ADI-R B). However, we did observe that girls had slightly fewer reported problems related to RRB (ADI-R C), which is in line with previous studies (Beggiato et al., 2017; Frazier et al., 2014; Supekar & Menon, 2015). The sex differences in RRB scores did not reach the adjusted significance level in our study (*p* = 0.038). Furthermore, others have found girls who meet the golden-standard diagnostic measures of ASD to be more severely affected than boys on parent-reported daily social skills and adaptive functioning and argue that standardized diagnostic tools like ADI-R might fail to detect all female autistic traits (Ratto et al., 2018).

The participants in our study did not significantly differ in the total amount of executive difficulties (GEC), but girls had higher scores (were slightly more impaired) than boys on the metacognitive index from the BRIEF. However, the sex differences in MI scores did not reach the adjusted significance level in our study (*p* = 0.045). White and colleagues (White et al., 2017) reported that girls showed more EF difficulties in a matched sample of 78 girls and 158 boys with an ASD diagnosis. The BRIEF (GEC) scores for girls and boys from their study are similar to our results; however, in our study the difference in GEC scores between girls and boys did not reach the corrected level of significance. This might be due to a smaller sample size and a stricter control for multiple testing in our study. The type of EF measurement, intelligence levels and age of the participant are probably also of importance and can explain inconsistent results when it comes to sex differences in EF.

We showed a positive association between EF deficits in everyday life and social dysfunction for girls with ASD. The main finding in our study is not that girls with ASD have more EF deficits than boys, but that the EF deficits have a stronger link to core ASD symptoms in girls. Our study only investigated the association between EF and social function, and does not provide insights into the causal relationship between these two characteristics.

In TD children, girls appear to be more mature than boys, better at adapting to the classroom environment, and more sociable (Bennett et al., 2005). These differences may explain why girls tend to outperform boys in the early school years (Bennett et al., 2005). Consequently, there tends to be different patterns of social relationships and societal expectations of girls and boys in terms of social functioning. Boys often play in larger groups and are more likely to focus on games with formal rules, while girls tend to form smaller more intimate groups and focus on conversations and reciprocal friendships. Since reciprocal social interaction and communication are the core challenges for people with ASD, girls with ASD might have more difficulties socially interacting with other girls, than boys with ASD have socially interacting with other boys because of different social patterns between how boys and girls tend to interact and play (Dean et al., 2014; Tierney et al., 2016). These more fluid and less structured activities that are characteristic of how girls interact socially, might be more dependent on well-functioning EF. Thus, when EF is impaired in girls with ASD, it may have stronger negative effects on their social functioning because it requires more of their total cognitive resources. It may be that males have several different pathways to the development of ASD than females, and that females that reach the threshold for an ASD diagnosis have less variation in their cognitive profiles. While males can probably reach the ASD threshold with varying degrees of EF problems, these cognitive challenges may be a more integrated part of ASD-related difficulties in females.

Although the ADI-R together with the ADOS is considered to be the gold standard for assessing ASD (Falkmer et al., 2013; Ozonoff et al., 2005), recent studies suggest that these diagnostic instruments may not be equally effective in identifying symptoms in both sexes. Beggiato et al. (2017) investigated if the ADI-R items discriminate between boys and girls and found that in two large cohorts the ADI-R was better at classifying boys than girls. They argue that because clinicians use diagnostic tools (like the ADOS and the ADI-R) that are not gender specific, it is likely that girls are underrepresented. Other screening instruments for autism symptoms like the autism spectrum screening questionnaire (ASSQ) and the social responsiveness scale (SRS) have gender-specific items or different norms for boys and girls, to better to capture the “female phenotype” of autism (Beggiato et al., 2017). Thus, although girls and boys in our study have the same level of difficulties in social reciprocity and communication, they might have different expressions of autism symptoms in everyday life. We did not use the screening tools ASSQ or SRS because ADI-R is considered the gold standard measure of autism symptomatology. Further, ADI-R involves a clinical rating and not just parent reports, taking into account the clinical judgment. However, it might be that the diagnostic instruments like the ADI-R are less sensitive to female ASD characteristics (Beggiato et al., 2017), and that females that get a ASD diagnosis often need to have additional cognitive or behavioral problems before receiving a referral to the clinic (Dworzynski et al., 2012). Even though the ADI-R have three domains, the two domains reciprocal social interaction and communication are collapsed together in DSM-5. Our finding that the two domains both were associated with EF deficits in girls but not boys, supports that the two areas reciprocal social interaction and communication are interconnected. Longitudinal studies have revealed that EF deficits in early childhood for people with ASD are prognostic for autistic features and adaptive function 12 years later (Kenny et al., 2019), and it might be possible to improve EF by interventions (Kenworthy et al., 2014). Therefore, it is important to identify if there are sex differences in the relationship between EF and ASD features.

In our study 34.5% of the children had a comorbid diagnosis of ADHD. Both ASD and ADHD are characterized by executive dysfunction, but the two disorders typically differ in terms of which subdomains of EF that are affected. Where individuals with ADHD usually have problems with inhibition, those with ASD are more likely to have difficulties with flexibility and planning (Craig et al., 2016). Recently, it was suggested that as many as 40–70% of children and adolescents with ASD have a comorbid diagnosis of ADHD (Antshel et al., 2016; Lai et al., 2014; Simonoff et al., 2008). This complicates the picture regarding EF deficits, considering that the two disorders typically represent different aspects of EF deficits. In our study we did not have any significant sex differences in the distribution of ADHD. Furthermore, we included ADHD diagnosis as a predictor in our nested regression models (Tables 2, 3 and 4). ADHD diagnosis did not have a significant contribution to the outcome measures related to social reciprocity, communication or RRB. We argue that it is important to include children and adolescents with comorbid ADHD in research on ASD, because ADHD is a common comorbid disorder in clinical populations. However, it is important to be aware of the possible influence ADHD might have on executive measures. Future research should combine the questionnaire and diagnostic interview used in this study with direct observation of autistic symptoms (ADOS-2), neuropsychological testing and/or genetic information to investigate sex differences in the relationship between EF and social difficulties in more depth.

### Potential Clinical Implications

The finding that executive dysfunction and social difficulties are highly related in girls but not in boys might be important for various aspects of clinical practice. Firstly, when girls present with high scores on the ADI-R, it is reasonable to assess for executive difficulties and vice versa. Furthermore, because girls might have a higher risk for executive dysfunction in combination with their social difficulties, the finding can have implications for the choice of interventions. Following this argument, it is possible that girls (with the same degree of social difficulties as boys) will benefit more from EF interventions. Some existing programs that aim to enhance EF have shown to be effective on both social problems and EF (Kenworthy et al., 2014). However, to our knowledge, research is yet to investigate whether this treatment may be more effective for girls than boys. Future studies need to consider that sex differences might influence the effect of interventions.

### Strengths and Limitations of the Study

The study recruited a clinically well-defined sample of children and adolescents with ASD, however, there were still relatively few girls included in the study. The participants were recruited from specialist health care services, which may limit the results to more severe conditions as previous studies have shown that girls referred to specialist clinics have more severe problems than boys (Wang et al., 2017). The girls in our study were slightly older than the boys, but age was accounted for in the nested linear models. The BRIEF is based on parent’s own observations and evaluations of the child. This parental bias might have influenced the findings, but on the other hand, this instrument has been shown to be an ecologically valid measurement of how the child functions in everyday life. We have used the *t*-score from the BRIEF in the analyses, which have age and gender “corrected” norms, since *t*-scores are commonly used in literature, as well as clinical practice, and it is important to understand how different clinical tools influences each other. As our study included a relatively small sample of females and a large age range, we also performed a FUZZY match analysis to investigate the phenomena between the females and males in a more closely matched sample on age and IQ. Similar findings from the FUZZY match sample and the total sample contributes to the validity of our results, even though we had a relatively small sample of females and a large age range. Both the BRIEF and the ADI-R are based on information from parents and this might bias the findings. However, while the BRIEF is a questionnaire, the ADI-R is a clinical semi-structured interview, which involves a clinical rating. Together, they both give important information about a child’s behavior. In future studies it would be interesting to investigate possible sex differences in the relationship between the semi structured observation ADOS, which is a direct measurement of the child’s behavior, and BRIEF scores. In this way, one can further investigate if our findings are related to actual sex differences or whether it is more an expression of different parental reporting based on sex. Unfortunately, this was not possible in the current study. We did not have access to ADOS item-level scores and therefore were not able to calculate comparable severity scores across the different ADOS versions and modules used in this study.

Another potential reason for the sex difference in ASD prevalence might be that girls have a different phenotype. Currently, the established diagnostic practices and tools like the ADOS and the ADI-R are not constructed or adapted to measure the subtle difficulties that girls may present with, which differ from the typical presentation of ASD symptoms in boys. Lai and colleagues suggest this might be a circular phenomenon, since an ASD diagnosis is based on behavioral descriptions, and the most common diagnostic tools are largely validated on the classic male phenotype of autism behaviors (Lai et al., 2015). Since we did not include TD children and adolescents in our study, we do not know if our findings are specifically related to ASD or if there is a more general sex difference between social dysfunction and EF which applies to TD children as well as other diagnostic groups.

## Conclusion

We report sex differences in the relationship between parent rated executive dysfunction and social difficulties in individuals with ASD. Our study found a significant relationship between difficulties with social reciprocity and communication and parent-rated executive dysfunction in girls, while the same relationship was not evident in boys. These results suggest potential underlying factors related to different manifestations of ASD in boys and girls, which may have clinical implications.

## Supplementary Information

Below is the link to the electronic supplementary material.

Supplementary file1 (PDF 335 kb)

## Data Availability

The datasets used and analyses in the current study are available from the corresponding author on reasonable request.
