# Supplementary material for: Sex as a Moderator Between Parent Ratings of Executive Dysfunction and Social Difficulties in Children and Adolescents with Autism Spectrum Disorder
Source: J Autism Dev Disord. 2022 Jul 15;53(10):3847–59. doi: 10.1007/s10803-022-05629-5 (PMC10499744; doi:10.1007/s10803-022-05629-5)
Supplement: Supplementary file 1 — Supplementary file1 (PDF 335 kb) [file 10803_2022_5629_MOESM1_ESM.pdf]

Supplementary Information

Sex as a moderator between parent ratings of executive dysfunction and social difficulties in children and adolescents with autism spectrum disorder

Tonje Torske [a,b*], Terje Nærland [c,d], Daniel S. Quintana [b,c,d,e], Ruth Elizabeth Hypher [f,m], Anett Kaale [c,g], Anne Lise Høyland [h,i], Sigrun Hope [e,j], Jarle Johannessen [c,k], Merete G Øie [b,l] and Ole A Andreassen [d,e]

[a] Division of Mental Health and Addiction, Vestre Viken Hospital Trust, Drammen, Norway

[b] Department of Psychology, University of Oslo, Norway

[c] NevSom Department of Rare Disorders and Disabilities, Oslo University Hospital, Oslo, Norway

[d] K.G. Jebsen Center for Neurodevelopmental Disorders, University of Oslo, Norway

[e] NORMENT, University of Oslo and Oslo University Hospital, Oslo, Norway

[f] Division of Mental Health and Addiction, Oslo University Hospital, Oslo, Norway

[g] Department of Special Needs Education, University of Oslo, Norway

[h] Regional Centre for Child and Youth Mental Health and Child Welfare, Department of Mental Health, Faculty of Medicine and Health Sciences, Norwegian University of Science and Technology, Trondheim, Norway

[i] Department of Pediatrics, St. Olav Hospital, Trondheim University Hospital, Trondheim, Norway

[j] Department of Neurohabilitation, Oslo University Hospital, Oslo, Norway

[k] Department of Medicine, University of Oslo, Norway

[l] Research Department, Innlandet Hospital Trust, Lillehammer, Norway

[m] Department of Clinical Neurosciences for Children, Division of Pediatric and Adolescent Medicine, Oslo University Hospital, Norway

**Corresponding author*
E-mail: tonje.torske@vestreviken.no (TT)

Supplementary Table S1. Age, IQ, BRIEF and ADI-R scores for girls and boys with ASD matched sample (N = 66) [a]

Scale	Girls		Boys		df	*p-value*	Glass' delta
	M (SD)	n	M (SD)	n			
Age	11.9 (3.1)	22	11.2 (3.3)	44	44.6	0.415	-0.21
Full-scale IQ	93.5 (9.3)	22	92.1 (11.3)	44	50.1	0.604	-0.12
BRIEF Global Executive Composite (GEC)	69.5 (10.5)	22	67.0 (11.3)	44	45.0	0.376	-0.22
BRIEF Behavioral Regulation Index (BRI)	68.3 (15.2)	22	67.8 (11.7)	42	34.6	0.891	-0.04
BRIEF Metacognition Index (MI)	68.4 (8.7)	22	64.2 (11.2)	44	52.6	0.102	-0.38
ADI-R (A) Reciprocal Social Interaction domain	11.7 (6.5)	22	12.1 (5.1)	44	34.4	0.842	0.08
ADI-R (B) Communication domain	8.9 (5.6)	21	9.3 (4.5)	43	33.2	0.803	0.09
ADI-R (C) Restricted, repetitive and stereotyped behavior domain	2.5 (2.1)	21	3.9 (2.4)	43	44.8	0.021	0.58

* $p = 0.008$
Welch's t-tests were conducted for age, IQ, BRIEF and ADI-R comparisons between sexes.

IQ = Intelligence Quotient
BRIEF: Behavior Rating Inventory of Executive Functions
ADI-R: Autism Diagnostic Interview-Revised diagnostic algorithm
Note. BRIEF scores are reported as T scores (M = 50, SD = 10) and ADI-R scores are reported as domain scores from the diagnostic algorithm.

[a] The participants were recruited from Norwegian health services specializing in the assessment of ASD and other neurodevelopmental disorders. The study was part of the national BUPGEN network. The matched sample consisted of 22 girls and 44 boys with ASD who were recruited between 2013 and May 2018 and assessed at age 5-19 years. Nine of the children (2 girls, 7 boys) were diagnosed with childhood autism, 5 (2 girls, 3 boys) with atypical autism, 27 (11 girls, 16 boys) with Asperger syndrome and 25 (7 girls, 18 boys) with unspecified pervasive developmental disorder (PDD-NOS). The male:female ratio was 2:1. In total, 17 children (25.8%) had a comorbid disorder of attention deficit/hyperactivity disorder (ADHD). Participants had an intelligence quotient (IQ) within the normal range based on a standardized Wechsler's test (Full-scale IQ ≥ 70) and spoke Norwegian fluently. Exclusion criteria were significant sensory losses (vision and/or hearing).

Supplementary Table S2. Nested hierarchical model summary: Reciprocal Social Interaction domain matched sample (N = 66)

ADI-R A		R^2	*B*	*SE B*	*p*
Model 1		0.03			0.740
	Constant		20.95	6.80	0 .003*
	Sex		-0.28	1.50	0.850
	IQ		-0.08	0.07	0.219
	ADHD diagnosis		-1.06	1.62	0.516
	Age		-0.06	0.22	0.783
Model 2		0.15			0.070
	Constant		8.89	7.62	0.248
	Sex		-0.79	1.42	0.580
	IQ		-0.07	0.06	0.275
	ADHD diagnosis		-1.65	1.54	0.288
	Age		-0.10	0.21	0.614
	BRIEF GEC		0.18	0.06	0.005*
Model 3		0.29			0.002*
	Constant		46.82	13.16	<0.001*
	Sex		-30.27	8.75	0.001*
	IQ		-0.07	0.06	0.203
	ADHD diagnosis		-0.43	1.46	0.768
	Age		-0.03	0.19	0.878
	BRIEF GEC		-0.39	0.18	0.031
	BRIEF GEC * Sex		0,43	0.13	0.001*

* p = 0.017
ADI-R: Autism Diagnostic Interview- Revised, diagnostic algorithm. A: Reciprocal Social Interaction domain, B: Communication domain, C: Restricted, repetitive and stereotyped behavior domain.
ADHD: Attention deficit/ hyperactivity disorder
IQ: Intelligence Quotient
BRIEF_GEC: Behavior Rating Inventory of Executive Function, Global Executive Composite
B = unstandardized regression coefficients

Supplementary Table S3. Nested hierarchical model summary: Communication domain matched sample (N = 66)

ADI-R B		R^2	*B*	*SE B*	*p*
Model 1		0.04			0.604
	Constant		16.87	5.89	0 .006*
	Sex		-0.10	1.31	0.938
	IQ		-0.08	0.06	0.180
	ADHD diagnosis		0.95	1.43	0.511
	Age		-0.05	0.19	0.802
Model 2		0.14			0.122
	Constant		7.85	6.72	0.248
	Sex		-0.56	1.27	0.662
	IQ		-0.07	0.06	0.239
	ADHD diagnosis		0.56	1.38	0.685
	Age		-0.09	0.19	0.614
	BRIEF GEC		0.13	0.05	0.016*
Model 3		0.27			0.004*
	Constant		40.29	11.68	0.001*
	Sex		-26.08	7.86	0.002*
	IQ		-0.07	0.05	0.195
	ADHD diagnosis		1.66	1.32	0.212
	Age		-0.05	0.17	0.764
	BRIEF GEC		-0.35	0.16	0.029
	BRIEF GEC * Sex		0.37	0.11	0.002*

* $p = 0.017$
ADI-R: Autism Diagnostic Interview- Revised, diagnostic algorithm. A: Reciprocal Social Interaction domain, B: Communication domain, C: Restricted, repetitive and stereotyped behavior domain.
ADHD: Attention deficit/ hyperactivity disorder
IQ: Intelligence Quotient
BRIEF_GEC: Behavior Rating Inventory of Executive Function, Global Executive Composite
B = unstandardized regression coefficients

Supplementary Table S4. Nested hierarchical model summary: Restricted, repetitive and stereotyped behavior domain matched sample (N = 66)

ADI-R C		R^2	*B*	*SE B*	*p*
Model 1		0.08			0.285
	Constant		6.40	2.86	0.029
	Sex		-1.38	0.64	0.034
	IQ		-0.01	0.03	0.690
	ADHD diagnosis		0.02	0.69	0.972
	Age		0.00	0.09	0.958
Model 2		0.16			0.067
	Constant		2.26	3.29	0.494
	Sex		-1.59	0.62	0.013*
	IQ		-0.01	0.03	0.835
	ADHD diagnosis		-0.15	0.67	0.823
	Age		-0.03	0.09	0.776
	BRIEF GEC		0.06	0.03	0.023
Model 3		0.19			0.048
	Constant		10.33	6.10	0.096
	Sex		-7.94	4.10	0.058
	IQ		-0.01	0.03	0.824
	ADHD diagnosis		0.12	0.69	0.859
	Age		-0.02	0.09	0.864
	BRIEF GEC		-0.06	0.08	0.472
	BRIEF GEC * Sex		0.09	0.06	0.123

* $p = 0.017$
ADI-R: Autism Diagnostic Interview- Revised, diagnostic algorithm. A: Reciprocal Social Interaction domain, B: Communication domain, C: Restricted, repetitive and stereotyped behavior domain.
ADHD: Attention deficit/ hyperactivity disorder
IQ: Intelligence Quotient
BRIEF_GEC: Behavior Rating Inventory of Executive Function, Global Executive Composite
B = unstandardized regression coefficients

Supplementary Table S5. Bootstrapped model ADI-R A

Model 1				
Predictors	Direct model		Bootstrapped model	
	2.50 %	97.50 %	2.50 %	97.50 %
Intercept	9.65	30.30	10.88	29.43
Sex	-2.75	2.49	-3.27	2.81
IQ	-0.16	0.02	-0.15	0.00
ADHD	-3.56	1.04	-3.59	1.14
Age	-0.42	0.25	-0.40	0.19

Model 2	Direct model		Bootstrapped model	
Predictors	2.50 %	97.50 %	2.50 %	97.50 %
Intercept	-1.14	22.49	-0.59	20.59
Sex	-3.09	1.99	-3.21	2.25
IQ	-0.15	0.01	-0.13	0.01
ADHD	-4.13	0.38	-4.30	0.60
Age	-0.40	0.25	-0.39	0.23
BRIEF GEC	0.04	0.24	0.05	0.25

Model 3				
Predictors	Direct model		Bootstrapped model	
	2.50 %	97.50 %	2.50 %	97.50 %
Intercept	22.99	66.39	26.46	62.74
Sex	-45.07	-13.34	-42.59	-14.55
IQ	-0.15	0.01	-0.14	0.00
ADHD	-3.21	1.14	-3.40	1.25
Age	-0.36	0.25	-0.34	0.25
BRIEF GEC	-0.66	-0.07	-0.63	-0.11
BRIEF GEC * Sex	0.19	0.65	0.21	0.61

ADI-R: Autism Diagnostic Interview- Revised, diagnostic algoritm. A: Reciprocal Social Interaction domain, B: Communication domain, C: Restricted, repetitive and steroetyped behavior domain
IQ: Intelligence Quotient
ADHD: Attention deficit/ hyperactivity disorder
BRIEF_GEC: Behavior Rating Inventory of Executive Function, Global Executive Composite

The standardized residuals from models 1 (p = 0.02), 2 (p = 0.01), and 3 (p = 0.003) were not normally distributed. Confidence intervals for the intercept and slopes of this model were similar to a bootstrapped model (Supplementary Table S5), indicating that there were no considerable problems with non-normal distribution of residuals in the model.

Supplementary Table S6. Bootstrapped model ADI-R B

Model 1				
Predictors	Direct model		Bootstrapped model	
	2.50 %	97.50 %	2.50 %	97.50 %
Intercept	3.98	21.74	4.34	21.20
Sex	-2.68	1.85	-2.82	2.31
IQ	-0.11	0.04	-0.10	0.04
ADHD	-1.57	2.45	-1.64	2.53
Age	-0.28	0.30	-0.23	0.27

Model 2				
Predictors	Direct model		Bootstrapped model	
	2.50 %	97.50 %	2.50 %	97.50 %
Intercept	-4.14	16.35	-3.40	14.52
Sex	-2.99	1.47	-2.98	1.70
IQ	-0.11	0.04	-0.10	0.03
ADHD	-2.01	1.98	-2.16	2.17
Age	-0.27	0.29	-0.28	0.28
BRIEF GEC	0.02	0.19	0.02	0.19

Model 3				
Predictors	Direct model		Bootstrapped model	
	2.50 %	97.50 %	2.50 %	97.50 %
Intercept	16.08	54.31	19.23	52.03
Sex	-39.47	-11.26	-38.80	-12.33
IQ	-0.10	0.04	-0.09	0.03
ADHD	-1.18	2.68	-1.27	2.76
Age	-0.25	0.28	-0.24	0.25
BRIEF GEC	-0.59	-0.07	-0.59	-0.09
BRIEF GEC * Sex	0.15	0.56	0.17	0.55

ADI-R: Autism Diagnostic Interview- Revised, diagnostic algorithm. A: Reciprocal Social Interaction domain, B: Communication domain, C: Restricted, repetitive and stereotyped behavior domain
IQ: Intelligence Quotient
ADHD: Attention deficit/ hyperactivity disorder
BRIEF_GEC: Behavior Rating Inventory of Executive Function, Global Executive Composite

The standardized residuals from models 1 (p = 0.004), 2 (p = 0.02), and 3 (p = 0.01) were not normally distributed. Confidence intervals for the intercept and slopes of this model were similar to a bootstrapped model (Supplementary Table S6), indicating that there were no considerable problems with non-normal distribution of residuals in the model.

Supplementary Table S7. Bootstrapped model ADI-R C

Model 1

Predictors	Direct model		Bootstrapped model	
	2.50 %	97.50 %	2.50 %	97.50 %
Intercept	1.41	9.98	1.78	9.67
Sex	-2.09	0.10	-2.05	0.10
IQ	-0.05	0.02	-0.05	0.02
ADHD	-1.35	0.57	-1.25	0.68
Age	-0.13	0.15	-0.11	0.13

Model 2

Predictors	Direct model		Bootstrapped model	
	2.50 %	97.50 %	2.50 %	97.50 %
Intercept	-2.28	7.65	-2.03	7.51
Sex	-2.23	-0.07	-2.14	-0.10
IQ	-0.05	0.02	-0.05	0.02
ADHD	-1.54	0.37	-1.47	0.49
Age	-0.13	0.15	-0.10	0.13
BRIEF GEC	0.01	0.09	0.00	0.09

Model 3

Predictors	Direct model		Bootstrapped model	
	2.50 %	97.50 %	2.50 %	97.50 %
Intercept	0.64	20.01	1.92	19.60
Sex	-14.77	-0.47	-14.28	-1.56
IQ	-0.05	0.02	-0.05	0.03
ADHD	-1.35	0.58	-1.30	0.64
Age	-0.12	0.15	-0.09	0.14
BRIEF GEC	-0.20	0.06	-0.19	0.05
BRIEF GEC * Sex	-0.01	0.20	0.00	0.19

ADI-R: Autism Diagnostic Interview- Revised, diagnostic algorithm. A: Reciprocal Social Interaction domain, B: Communication domain, C: Restricted, repetitive and stereotyped behavior domain
IQ: Intelligence Quotient
ADHD: Attention deficit/ hyperactivity disorder
BRIEF_GEC: Behavior Rating Inventory of Executive Function, Global Executive Composite

The standardized residuals from models 2 and 3, which included the predictor of EF were normally distributed ($p > 0.05$), however, they were not normally distributed for the first model ($p = 0.01$). Confidence intervals for the intercept and slopes of this model were similar to a bootstrapped model (Supplementary Table S7), indicating that there were no considerable problems with non-normal distribution of residuals in the model.

Supplementary Table S8. Age, IQ, BRIEF and ADI-R scores for girls and boys with ASD age 11 and younger (pre-puberty) (N=70)

Scale	Girls		Boys		df	*p-value*	Glass' delta
	Mean (SD)	n	Mean (SD)	n			
Age	9.3 (1.7)	11	8.5 (2.0)	59	16.1	0.219	-0.44
Full-scale IQ	94.6 (8.8)	10	97.9 (12.4)	49	17.2	0.334	0.37
BRIEF Global Executive Composite (GEC)	70.4 (9.3)	11	66.5 (11.2)	59	23.2	0.240	-0.42
BRIEF Behavioral Regulation Index (BRI)	68.5 (16.4)	11	67.5 (11.6)	54	12.1	0.841	-0.06
BRIEF Metacognition Index (MI)	69.4 (6.5)	11	63.8 (11.8)	59	24.2	0.035	-0.86
ADI-R (A) Reciprocal Social Interaction domain	13.5 (5.5)	11	11.7 (4.9)	59	13.2	0.347	-0.32
ADI-R (B) Communication domain	8.6 (4.5)	11	9.4 (4.5)	57	14.1	0.619	0.17
ADI-R (C) Restricted, repetitive and stereotyped behavior domain	2.4 (2.5)	11	3.3 (2.1)	58	12.9	0.244	0.39

* p = 0.008
Welch's t-tests were conducted for age, IQ, BRIEF and ADI-R comparisons between sexes

IQ = Intelligence Quotient
BRIEF: Behavior Rating Inventory of Executive Functions
ADI-R: Autism Diagnostic Interview-Revised

Note. BRIEF scores are reported as T scores (M = 50, SD = 10) and ADI-R scores are reported as domain scores from the diagnostic algorithm.

Supplementary Table S9. Age, IQ, BRIEF and ADI-R scores for girls and boys with ASD age 12 and older (post-puberty) (N=46)

Scale	Girls<br>Mean (SD)	n	Boys<br>Mean (SD)	n	df	*p-value*	Glass' delta
Age	14.1 (2.1)	14	13.8 (1.8)	32	21.4	0.613	-0.16
Full-scale IQ	92.5 (10.0)	12	92.0 (13.6)	31	27.5	0.902	-0.05
BRIEF<br>Global Executive Composite (GEC)	68.6 (11.1)	14	68.5 (10.2)	32	23.2	0.960	-0.02
BRIEF<br>Behavioral Regulation Index (BRI)	66.9 (13.7)	14	68.8 (12.4)	32	22.8	0.662	0.14
BRIEF Metacognition Index (MI)	68.1 (9.6)	14	65.8 (9.4)	32	24.3	0.473	-0.23
ADI-R (A)<br>Reciprocal Social Interaction domain	10.6 (6.5)	14	11.8 (5.4)	32	21.1	0.551	0.18
ADI-R (B)<br>Communication domain	8.9 (5.9)	13	8.9 (4.1)	30	17.3	0.989	-0.00
ADI-R (C)<br>Restricted, repetitive and stereotyped behavior domain	2.4 (1.7)	13	3.5 (2.4)	30	31.19	0.083	0.67

* $p = 0.008$
Welch's t-tests were conducted for age, IQ, BRIEF and ADI-R comparisons between sexes

IQ = Intelligence Quotient
BRIEF: Behavior Rating Inventory of Executive Functions
ADI-R: Autism Diagnostic Interview-Revised

Note. BRIEF scores are reported as T scores (M = 50, SD = 10) and ADI-R scores are reported as domain scores from the diagnostic algorithm.

Supplementary Table S10. Nested hierarchical model summary: Reciprocal social interaction domain and Behavior Regulation Index

ADI-R A		R^2	B	SE B	95% CI	*p*
Model 1		0.03				0.486
	Constant		19.98	5.20	[9.65, 30.30]	< .001*
	Sex		-0.13	1.32	[-2.75, 2.49]	0.923
	IQ		-0.07	0.04	[-0.16, 0.02]	0.104
	ADHD diagnosis		-1.26	1.16	[-3.56, 1.04]	0.279
	Age		-0.08	0.17	[-0.42, 0.25]	0.618
Model 2		0.14				0.015*
	Constant		13.93	5.81	[2.38, 25.47]	0.019
	Sex		-0.31	1.26	[-2.83, 2.21]	0.807
	IQ		-0.10	0.04	[-0.18, -0.01]	0.369
	ADHD diagnosis		-2.23	1.15	[-4.52, 0.06]	0.056
	Age		-0.09	0.17	[-0.44, 0.27]	0.631
	BRIEF BRI		0.13	0.04	[0.04, 0.21]	0.003*
Model 3		0.21				0.001*
	Constant		34.76	9.29	[16.30, 53.21]	< .001*
	Sex		-17.32	6.17	[-29.58, -5.06]	0.006*
	IQ		-0.08	0.04	[-0.17, 0.00]	0.055
	ADHD diagnosis		-1.43	1.15	[-3.71, 0.85]	0.215
	Age		-0.04	0.17	[-0.38, 0.30]	0.819
	BRIEF BRI		-0.21	0.13	[-0.46, 0.04]	0.104
	BRIEF BRI * Sex		0.25	0.09	[0.07, 0.43]	0.006*

$p = 0.017$

ADI-R: Autism Diagnostic Interview- Revised, diagnostic algorithm. A: Reciprocal Social Interaction domain, B: Communication domain, C: Restricted, repetitive and stereotyped behavior domain.
ADHD: Attention deficit/ hyperactivity disorder
IQ: Intelligence Quotient
BRIEF_BRI: Behavior Rating Inventory of Executive Function, Behavioral Regulation Index
B = unstandardized regression coefficients, CI = confidence interval

Supplementary Table S11. Nested hierarchical model summary: Communication domain and Behavior Regulation Index

ADI-R B		R^2	B	SE B	95% CI	*p*
Model 1		0.01				0.843
	Constant		12.86	4.47	[3.98, 21.74]	0 .005*
	Sex		-0.42	1.14	[-2.68, 1.85]	0.717
	IQ		-0.04	0.04	[-0.11, 0.04]	0.357
	ADHD diagnosis		0.44	1.01	[-1.57, 2.45]	0.664
	Age		0.01	0.15	[-0.28, 0.30]	0.963
Model 2		0.09				0.129
	Constant		7.81	5.11	[-2.34, 17.97]	0.130
	Sex		-0.58	1.12	[-2.81, 1.66]	0.609
	IQ		-0.04	0.04	[-0.12, 0.04]	0.278
	ADHD diagnosis		0.17	1.03	[-1.88, 2.22]	0.871
	Age		-0.06	0.16	[-0.37, 0.25]	0.709
	BRIEF BRI		0.10	0.04	[0.03, 0.17]	0.008*
Model 3		0.19				0.006*
	Constant		28.53	8.14	[12.35, 44.70]	0.001*
	Sex		-17.58	5.46	[-28.43, -6.72]	0.002*
	IQ		-0.03	0.03	[-0.11, 0.04]	0.416
	ADHD diagnosis		0.99	1.02	[-1.03, 3.01]	0.332
	Age		-0.03	0.15	[-0.33, 0.27]	0.850
	BRIEF BRI		-0.23	0.11	[-0.45, -0.01]	0.040
	BRIEF BRI * Sex		0.25	0.08	[0.09, 0.41]	0.002*

$p = 0.017$

ADI-R: Autism Diagnostic Interview- Revised, diagnostic algorithm. A: Reciprocal Social Interaction domain, B: Communication domain, C: Restricted, repetitive and stereotyped behavior domain.
ADHD: Attention deficit/ hyperactivity disorder
IQ: Intelligence Quotient
BRIEF_BRI: Behavior Rating Inventory of Executive Function, Behavioral Regulation Index
B = unstandardized regression coefficients, CI = confidence interval

Supplementary Table S12. Nested hierarchical model summary: Restricted, repetitive and stereotyped behavior domain and Behavior Regulation Index

ADI-R C		R^2	B	SE B	95% CI	*p*
Model 1		0.04				0.435
	Constant		5.70	2.16	[1.41, 9.98]	0 .010*
	Sex		-1.00	0.55	[-2.09, 0.10]	0.073
	IQ		-0.01	0.02	[-0.05, 0.02]	0.478
	ADHD diagnosis		-0.39	0.48	[-1.35, 0.57]	0.422
	Age		0.01	0.07	[-0.13, 0.15]	0.889
Model 2		0.08				0.211
	Constant		3.43	2.51	[-1.55, 8.42]	0.175
	Sex		-1.07	0.55	[-2.16, -0.03]	0.057
	IQ		-0.01	0.02	[-0.05, 0.03]	0.687
	ADHD diagnosis		-0.52	0.50	[-1.52, 0.47]	0.301
	Age		-0.02	0.08	[-0.17, 0.13]	0.804
	BRIEF BRI		0.03	0.02	[-0.00, 0.07]	0.072
Model 3		0.11				0.109
	Constant		9.46	4.14	[1.23, 17.70]	0.025
	Sex		-6.01	2.78	[-11.54, -0.49]	0.033
	IQ		-0.00	0.02	[-0.04, 0.03]	0.822
	ADHD diagnosis		-0.29	0.51	[-1.30, 0.73]	0.575
	Age		-0.01	0.08	[-0.16, 0.14]	0.893
	BRIEF BRI		-0.06	0.06	[-0.17, 0.05]	0.263
	BRIEF BRI * Sex		0.07	0.04	[-0.01, 0.15]	0.073

* $p = 0.017$

ADI-R: Autism Diagnostic Interview- Revised, diagnostic algorithm. A: Reciprocal Social Interaction domain, B: Communication domain, C: Restricted, repetitive and stereotyped behavior domain.
ADHD: Attention deficit/ hyperactivity disorder
IQ: Intelligence Quotient
BRIEF_BRI: Behavior Rating Inventory of Executive Function, Behavioral Regulation Index
B = unstandardized regression coefficients, CI = confidence interval

Supplementary Table S13. Nested hierarchical model summary: Reciprocal social interaction domain and Metacognition Index

ADI-R A		R^2	B	SE B	95% CI	*p*
Model 1		0.03				0.486
	Constant		19.98	5.20	[9.65, 30.30]	< .001*
	Sex		-0.13	1.32	[-2.75, 2.49]	0.923
	IQ		-0.07	0.04	[-0.16, 0.02]	0.104
	ADHD diagnosis		-1.26	1.16	[-3.56, 1.04]	0.279
	Age		-0.08	0.17	[-0.42, 0.25]	0.618
Model 2		0.09				0.111
	Constant		12.32	6.03	[0.35, 24.28]	0.044
	Sex		-0.70	1.31	[-3.30, 1.90]	0.594
	IQ		-0.07	0.04	[-0.15, 0.02]	0.121
	ADHD diagnosis		-1.87	1.16	[-4.18, 0.43]	0.110
	Age		-0.07	0.17	[-0.40, 0.26]	0.680
	BRIEF MI		0.12	0.05	[0.02, 0.22]	0.020
Model 3		0.18				0.004*
	Constant		47.07	12.00	[23.24, 70.90]	< .001*
	Sex		-30.98	9.27	[-49.38, -12.58]	0.001*
	IQ		-0.07	0.04	[-0.15, 0.01]	0.097
	ADHD diagnosis		-1.40	1.11	[-3.62, 0.81]	0.211
	Age		-0.07	0.16	[-0.38, 0.24]	0.656
	BRIEF MI		-0.40	0.16	[-0.72, -0.07]	0.018
	BRIEF MI * Sex		0.45	0.14	[0.18, 0.72]	0.001*

p = 0.017

ADI-R: Autism Diagnostic Interview- Revised, diagnostic algorithm. A: Reciprocal Social Interaction domain, B: Communication domain, C: Restricted, repetitive and stereotyped behavior domain.
ADHD: Attention deficit/ hyperactivity disorder
IQ: Intelligence Quotient
BRIEF_MI: Behavior Rating Inventory of Executive Function, Metacognition Index
B = unstandardized regression coefficients, CI = confidence interval

Supplementary Table S14. Nested hierarchical model summary: Communication domain and Metacognition Index

ADI-R B		R^2	B	SE B	95% CI	*p*
Model 1		0.01				0.843
	Constant		12.86	4.47	[3.98, 21.74]	0 .005*
	Sex		-0.42	1.14	[-2.68, 1.85]	0.717
	IQ		-0.04	0.04	[-0.11, 0.04]	0.357
	ADHD diagnosis		0.44	1.01	[-1.57, 2.45]	0.664
	Age		0.01	0.15	[-0.28, 0.30]	0.963
Model 2		0.06				0.328
	Constant		7.01	5.20	[-3.31, 17.34]	0.181
	Sex		-0.87	1.14	[-3.14, 1.40]	0.447
	IQ		-0.03	0.04	[-0.11, 0.04]	0.408
	ADHD diagnosis		-0.05	1.02	[-2.08, 1.97]	0.990
	Age		0.02	0.14	[-0.27, 0.30]	0.907
	BRIEF MI		0.09	0.04	[0.01, 0.18]	0.038
Model 3		0.13				0.047
	Constant		31.95	10.62	[10.85, 53.06]	0.003*
	Sex		-22.59	8.22	[-38.92, -6.26]	0.007*
	IQ		-0.03	0.04	[-0.10, 0.04]	0.383
	ADHD diagnosis		0.32	1.00	[-1.66, 2.30]	0.750
	Age		0.01	0.14	[-0.27, 0.28]	0.966
	BRIEF MI		-0.28	0.14	[-0.56, 0.01]	0.059
	BRIEF MI * Sex		0.32	0.12	[0.08, 0.56]	0.009*

$p = 0.017$

ADI-R: Autism Diagnostic Interview- Revised, diagnostic algorithm. A: Reciprocal Social Interaction domain, B: Communication domain, C: Restricted, repetitive and stereotyped behavior domain.
ADHD: Attention deficit/ hyperactivity disorder
IQ: Intelligence Quotient
BRIEF_MI: Behavior Rating Inventory of Executive Function, Metacognition Index
B = unstandardized regression coefficients, CI = confidence interval

Supplementary Table S15. Nested hierarchical model summary: Restricted, repetitive and stereotyped behavior domain and Metacognition Index

ADI-R C		R^2	B	SE B	95% CI	*p*
Model 1		0.04				0.435
	Constant		5.70	2.16	[1.41, 9.98]	0 .010*
	Sex		-1.00	0.55	[-2.09, 0.10]	0.073
	IQ		-0.01	0.02	[-0.05, 0.02]	0.478
	ADHD diagnosis		-0.39	0.48	[-1.35, 0.57]	0.422
	Age		0.01	0.07	[-0.13, 0.15]	0.889
Model 2		0.08				0.153
	Constant		2.91	2.51	[-2.28, 7.65]	0.249
	Sex		-1.22	0.55	[-2.23, -0.07]	0.030
	IQ		-0.01	0.02	[-0.05, 0.02]	0.540
	ADHD diagnosis		-0.62	0.49	[-1.54, 0.37]	0.210
	Age		0.01	0.07	[-0.13, 0.15]	0.832
	BRIEF MI		0.04	0.02	[0.01, 0.09]	0.040
Model 3		0.10				0.128
	Constant		9.28	5.27	[-1.18, 19.74]	0.081
	Sex		-6.77	4.08	[-14.87, 1.33]	0.100
	IQ		-0.01	0.02	[-0.05, 0.02]	0.533
	ADHD diagnosis		-0.52	0.49	[-1.50, 0.45]	0.287
	Age		0.01	0.07	[-1.13, 0.15]	0.862
	BRIEF MI		-0.05	0.07	[-0.19, 0.09]	0.486
	BRIEF MI * Sex		0.08	0.06	[-0.04, 0.20]	0.173

* $p = 0.017$

ADI-R: Autism Diagnostic Interview- Revised, diagnostic algorithm. A: Reciprocal Social Interaction domain, B: Communication domain, C: Restricted, repetitive and stereotyped behavior domain.
ADHD: Attention deficit/ hyperactivity disorder
IQ: Intelligence Quotient
BRIEF_MI: Behavior Rating Inventory of Executive Function, Metacognition Index
B = unstandardized regression coefficients, CI = confidence interval
